# Correlation of skull vibration-induced nystagmus test and video head impulse test in patients with sudden sensorineural hearing loss with vertigo

**DOI:** 10.3389/fneur.2025.1642700

**Published:** 2025-09-18

**Authors:** Qiang Guo, Ying Lin, Pengfei Hang, Dingjun Zha

**Affiliations:** Department of Otolaryngology, Head and Neck Surgery, Xijing Hospital, Air Force Medical University, Xi’an, China

**Keywords:** skull vibration-induced nystagmus test, sudden sensorineural hearing loss, hearing audiogram, video head impulse test, caloric test

## Abstract

**Objective:**

The study aims to examine the agreement between the skull vibration-induced nystagmus test (SVINT), video head impulse test (vHIT), and caloric test (CaT) in detecting vestibular function asymmetry in patients with unilateral sudden sensorineural hearing loss with vertigo (SSNHL-V).

**Methods:**

This study included 71 patients with SSNHL-V and 20 healthy controls. All participants underwent comprehensive audiological and vestibular function assessments. This study evaluated the correlation between SVINT and CaT/vHIT in detecting vestibular asymmetry. Furthermore, we analyzed the correlation between SVINT findings and (1) the classification of audiograms, and (2) the degree of hearing loss in SSNHL patients with vertigo.

**Results:**

The agreement between the result of SVINT and horizontal semicircular canal (HSCC) results of vHIT (*kappa* = 0.668, *p* < 0.05) was superior to that between the SVINT and CaT (*kappa* = 0.324, *p* < 0.05), as well as between the SVINT and vertical semicircular canal (SCC) results of vHIT (*kappa* = 0.345, *p* < 0.05). SVINT had a sensitivity of 96.7% and a specificity of 73.2% when using the HSCC results of vHIT as the standard. The SVINT did not correlate with the classification of the audiogram and the degree of hearing loss (*F* = 5.968, *p* > 0.05; *χ^2^* = 0.017, *p* > 0.05).

**Conclusion:**

Skull vibration-induced nystagmus test is a bedside test that enables simple and rapid screening for a high-frequency functional asymmetry of HSCC in patients with sudden sensorineural hearing loss with vertigo.

## 1 Introduction

Sudden sensorineural hearing loss (SSNHL) is clinically characterized by sensorineural hearing loss of ≥30 dB HL in at least three consecutive hearing frequencies occurring within 72 h (1). Vestibular involvement in patients with SSNHL was first reported in 1949 (2). Due to the embryological and anatomical correlation between the cochlea and the vestibular system, 30 to 40% of patients experience hearing loss accompanied by vestibular dysfunction, resulting in dizziness, vomiting, instability, and other clinical symptoms. Extensive studies have been conducted on the role of CaT, vHIT, and sensory organization tests (SOT) in assessing vestibular impairment and hearing recovery in patients with SSNHL (3–5). However, acute audiovestibular dysfunction may also result from central arterial occlusive disease, particularly the anterior inferior cerebellar artery (AICA) infarction. It has been reported that hearing loss is present in 60 to 90% of patients with AICA infarction (6, 7). HINTS Plus (head impulse, nystagmus, test of skew deviation, and bedside hearing screening) test battery, along with head MRI, facilitates diagnosis. The bedside vestibular test has a high degree of accuracy when performed by a trained physician. It offers the potential to reduce misdiagnosis while simultaneously decreasing diagnostic test overuse, unnecessary hospitalization, and incorrect treatments (8).

The vibration-induced nystagmus phenomenon was serendipitously observed in 1973 and gradually applied in clinical practice in 1999 (9). It was formally described as an independent vestibular function test at the 2006 International Society of Otoneurology (SIO) meeting in France (10). Based on currently reported experiments in guinea pigs (11, 12) and clinical studies (13), it is believed that vibration-induced nystagmus mainly originates from the cranial vibration in the cerebrospinal fluid that causes pressure waves to be transmitted to the endolymph, which results in mechanical shock stimulation of the vestibular receptors and causes the deflection of the hair cell stereocilia (14, 15). Bone-conducted vibration (BCV) exhibits greater selectivity for the otolith organs at 500 Hz (16) and activates both the semicircular canals and the otolith organs at frequencies ranging from 100 to 200 Hz (12). In healthy individuals with intact labyrinths on both sides, the stimulus produces the same neural response in both labyrinths and does not induce nystagmus. In patients with unilateral vestibular impairment, the asymmetry in neuronal response to stimulation in both labyrinths induces a predominantly horizontal nystagmus, with the fast phase beating away from the lesion side. The SVINT has received attention for its simplicity, efficiency, and non-invasiveness. It effectively responds to vestibular asymmetries on either side in peripheral diseases and has been described as the “vestibular Weber test.”

The first aim of this paper is to describe the results of using the vHIT, CaT, and SVINT to assess vestibular impairment in SSNHL-V groups. Next, it analyses the agreement of SVINT with CaT and vHIT in detecting vestibular asymmetry. Finally, the correlation between the SVINT, the classification of the audiogram, and the degree of hearing impairment in patients with SSNHL-V was analyzed, and the usefulness of the SVINT as a rapid bedside test in assessing vestibular asymmetry in patients with SSNHL-V was investigated.

## 2 Materials and procedure

### 2.1 Participants

This study was conducted in the Department of Otolaryngology and Head and Neck Surgery at a tertiary care hospital from 2022 to 2024. Patients with complaints of unilateral SSNHL-V were selected and underwent pure tone audiometry test, acoustic immittance test, vHIT, CaT, and SVINT on the same day. The pure tone average (PTA) in normal ears is ≤30 dB HL in patients, and the tympanogram of both ears is normal. All patients had not taken central nervous system (CNS) depressants or vigilance-influencing drugs within 48 h before testing. The inclusion criteria were (1) acute onset of sensorineural hearing loss at three consecutive frequencies ≥ 30 dB HL within 72 h; (2) with no clear etiology; (3) an acute attack of vertigo within 24 h of the onset of hearing loss. Exclusion criteria were (1) patients with spontaneous nystagmus, gaze-evoked nystagmus, oculomotor paralysis, history of other central nervous system disorders; (2) patients could not stand or walk without assistance; (3) patients with vertigo symptoms before the onset of SSNHL, tympanic membrane perforation, middle ear infections, or a history of previous middle ear surgery, as well as patients with concomitant vestibular disorders (Meniere’s disease, Ramsay-Hunt syndrome, Benign Paroxysmal Positional Vertigo etc.); (4) patients with abnormalities of saccade test, smooth pursuit test, and optokinetic nystagmus tests; (5) patients with retrocochlear lesion or AICA infarction in the MRI.

### 2.2 Skull vibration-induced nystagmus test

**Figure 1:**
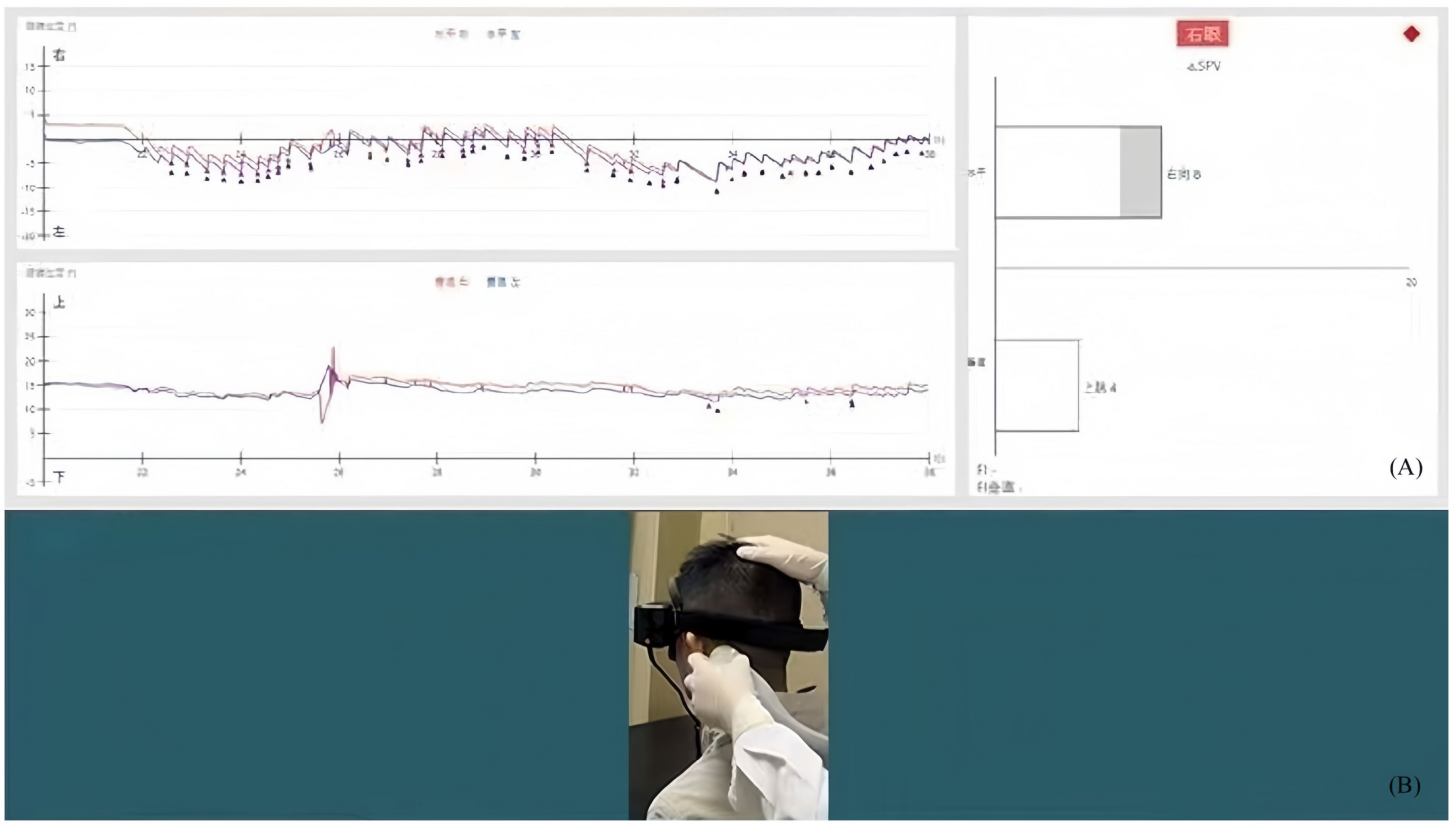
Nystagmus acquisition for the SVINT. **(A)** Recording of nystagmus: the red curve traces the right eye and the blue curve traces the left eye; the patient induced a horizontal rightward nystagmus. **(B)** Mastoid stimulation: the examiner is behind the subject; the other hand immobilizes the head.

Subjects were seated, wearing goggles equipped with a video camera, and deprived of visual fixation suppression. The examiner holds the subject’s head and places the vibrator (WAHL, America) vertically on the subject’s mastoid process, which is level with the external auditory canal. A 10 N or 1 kg (9) pressure was applied to make the vibrator press against the subject’s scalp. Nystagmus was observed with a video camera, and slow-phase eye velocity (SPV) data were recorded. Three stimulations were performed for each mastoid process with a stimulation frequency of 120 Hz. The duration of each stimulation was approximately 10 to 15 s. Recorded nystagmus was stimulus-locked, and five or more consecutive horizontal nystagmus with an SPV > 3°/s were considered positive (17) (Figure 1).

### 2.3 vHIT

**Figure 2:**
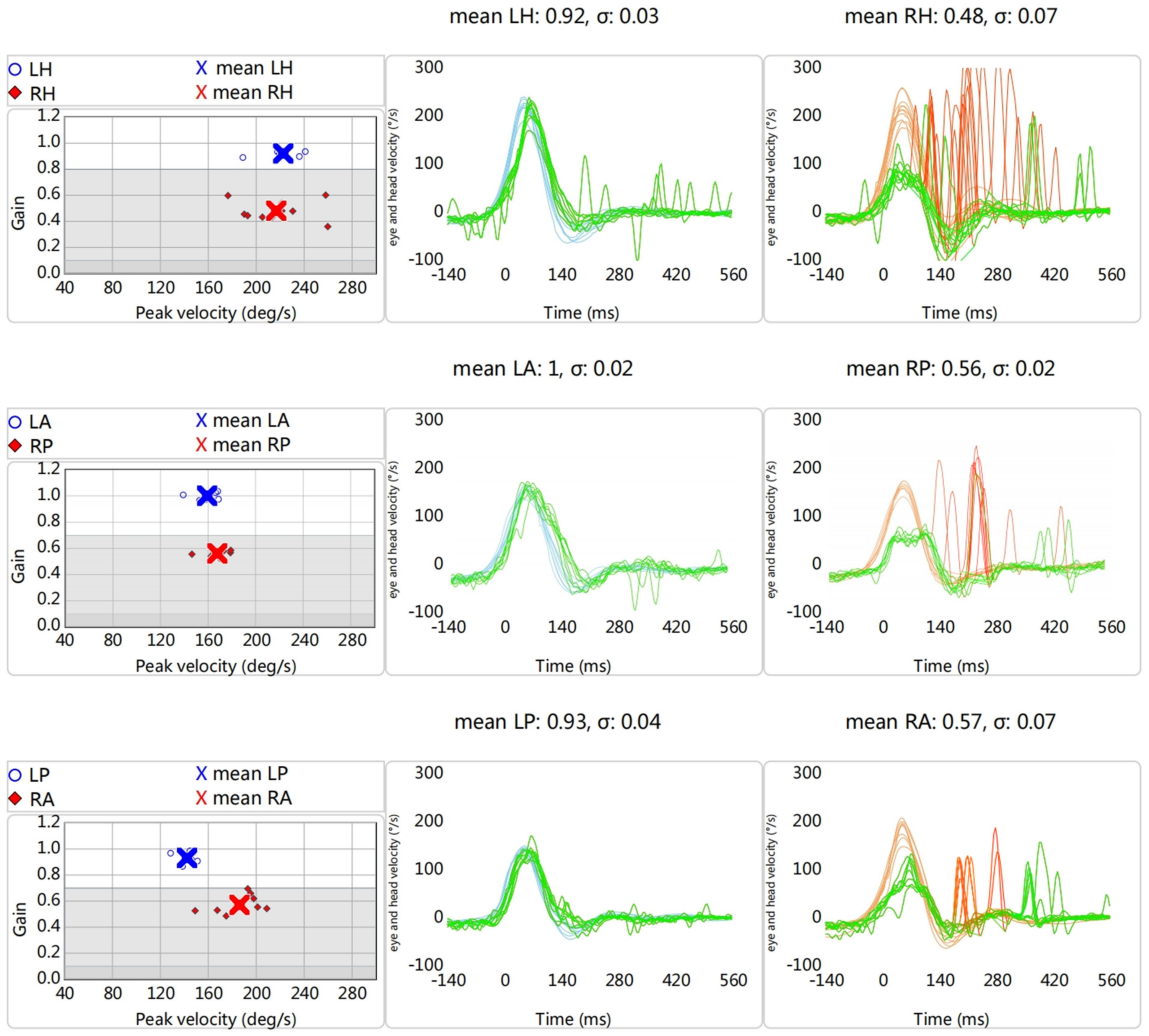
A patient with a right-side unilateral vestibular dysfunction; eye velocity (green line), head velocity (blue line indicates leftward head impulse; Orange line indicates rightward head impulse), catch-up saccades (red line); LH and RH, left horizontal and right horizontal head impulse; LA, left anterior head impulse; PR, right posterior head impulse; LP, left posterior head impulse; RA, right anterior head impulse.

In a brightly lit room, the subject wearing the device (CS Impulse, GN Otometrics Inc., Denmark) sits in front of a fixation target at a distance of 1.2 meters. Subjects were instructed to keep their eyes on the target during the test. The examiner gives the subject a small, unexpected, abrupt head turn. The device records the stimulus of passive head movement velocity (amplitude: 15–20°, peak velocity: 150–300°/s) and the response of ocular movement velocity (18). In healthy subjects, ocular movements can compensate for the sudden, erratic, and passive head rotation. The vestibulo-ocular reflex (VOR) mechanism allows the velocity of ocular movement to be equal to the head movement. However, the direction of ocular movement is opposite to the head movement. It enables stabilization of the target to the retina and maintains visual clarity. Ocular movements in patients with unilateral vestibular impairment do not effectively compensate for head movements when turning the head to the affected side. The eye follows the head movements and is unable to stay on target. To capture the target, the patient produces catch-up saccades (CuS) during or at the end of head movement. The area under the curve (AUC) of the eye velocity is divided by the AUC of the head velocity. VOR gains for the vertical SCC and the HSCC threshold were 0.7 and 0.8, respectively (19). VOR gain reduction with CuS is considered a positive result. Only reduced VOR gain without CuS in vHIT results were excluded because examiners’ operational errors or poor patient cooperation could not be ruled out (Figure 2).

### 2.4 Caloric test

The test was conducted in a dark room, and the patient wore goggles (Micromedical Technology, MMT) to record nystagmus. The patients had their heads tilted at 30° in the supine position, orienting the HSCC vertically. Cold (24°C) and warm (48°C) air were used to irrigate both ears for 1 min each in turn, with an interval of 5–7 min between each irrigation. Following each irrigation, the maximum SPV of the nystagmus was recorded, and the parameters of canal paresis were obtained. A CP > 25% suggested unilateral HSCC weaknesses at low frequencies (20). A bithermal SPV < 6°/sec on both sides indicated bilateral low-frequency hypofunction of HSCC (21).

### 2.5 Pure tone audiometry test

We calculated the pure-tone average (PTA) using thresholds at four frequencies: 0.5, 1, 2, and 4 kHz. Audiometry test results were classified into four conditions according to the guidelines of the Chinese Medical Association for SSNHL. Low-frequency hearing impairment type: ≥20 dB HL at least in frequencies ≤ 1 kHz. Flat hearing loss type: hearing loss at all frequencies (0.125, 0.5, 1, 2, 4, and 8 kHz), with a PTA ≤ 80 dB HL. High-frequency hearing loss type: hearing loss ≥ 20 dB HL at least in frequencies ≥ 2 kHz. Full-frequency hearing loss type: hearing loss at all frequencies (0.125, 0.5, 1, 2, 4, and 8 kHz), with a PTA ≥ 81 dB HL. The degree of hearing impairment was classified as mild (26–40 dB HL), moderate (41–60 dB HL), severe (61–80 dB HL), and profound (≥81 dB HL) hearing loss based on the results of PTA.

### 2.6 Statistical methods

SPSS 26.0 software was applied to process the data. Age comparisons between groups were performed using an independent sample *t*-test. Gender comparisons between groups were performed using the chi-square test. Fisher’s exact test (F) was used for vHIT intergroup comparisons. The McNemar test is used to evaluate whether there is a difference in the positive rates of two testing methods by analyzing the discordant data in the contingency table. The Kappa test assesses the agreement between two testing methods, incorporating all data in the contingency table during analysis (kappa values: ≤0.2, slight agreement; 0.21–0.4, fair agreement; 0.41–0.6, moderate agreement; 0.61–0.8, substantial agreement; 0.81–1, almost perfect agreement). Two test methods were used in combination. Use the Kappa test to assess the agreement of the two methods. If the agreement was low (kappa value < 0.4), the McNemar test was not meaningful. If the agreement was moderate, the McNemar test was used to supplement the analysis of whether there was a difference in the positive rates between the two methods. The statistical significance was considered at *p* < 0.05.

## 3 Results

### 3.1 Participant characteristics

Seventy-one patients were included in the SSNHL-V group, comprising 29 males and 42 females aged 14 to 74 years, with a mean age of 48.6 ± 13.4 years. 49.3% of the patients had an SSNHL on the left side (Table 1). Twenty healthy subjects, comprising 12 males and 8 females, had a mean age of 50.6 ± 13.3 years. The difference in age (*t* = 0.564, *p* = 0.821) and gender (*χ^2^* = 2.313, *p* = 0.128) between the two groups was not statistically significant. The data analysis excluded one case with down-beating nystagmus induced by SVINT.

**Table 1:** Demographic characteristics of the included SSNHL-V group.

Variable	Patients (*n* = 71)
Sex	
Male/Female	29 (40.8%)/42 (59.2%)
Age (years)	48.6 ± 13.4
Affected side	
Left/right	35 (49.3%)/36 (50.7%)
Hearing loss type	
Mild hearing loss (26–40 dB HL)	3 (4.2%)
Moderate hearing loss (41–60 dB HL)	8 (11.3%)
Severe hearing loss (61–80 dB HL)	21 (29.6%)
Profound hearing loss (≥ 81 dB HL)	39 (54.9%)
Audiogram type	
High-frequency hearing loss	8 (11.3%)
Low-frequency hearing loss	1 (1.4%)
Full-frequency hearing loss	36 (50.7%)
Flat-hearing loss	26 (36.6%)
Vestibular test results	
Positive CaT (unilateral and bilateral hypofunction)	55 (77.5%)
Positive vHIT	44 (62%)
Positive SVINT	40 (56.3%)

The SVINT, CaT, and vHIT results were normal in the control group. In the SSNHL-V group, 44 patients (62%) had positive vHIT results that showed a reduction in VOR gain combined with CuS in one or more SCCs on the affected side. Forty-seven patients (66.2%) had CaT results that showed unilateral hypofunction, and 8 patients (11.3%) had bilateral hypofunction. Forty patients (56.3%) had positive SVINT results, triggering horizontal nystagmus beating toward the healthy side. In three of these cases, the SPV on the affected side induced by SVINT was stronger than the SPV on the healthy side.

### 3.2 SVINT vs. CaT

Thirty-two patients (68.1%) with unilateral hypofunction of CaT had a positive SVINT result. Fifteen patients (31.9%) with unilateral hypofunction of CaT had a negative SVINT result, which included four vHIT results that showed reduced VOR gain combined with CuS in the PSCC on the affected side, one vHIT result that showed reduced VOR gain combined with CuS in the bilateral PSCC, and the remaining nine vHIT results were normal. Two of the eight patients with bilateral hypofunction of CaT showed positive SVINT results. In one case, vHIT results showed reduced VOR gain combined with CuS in the HSCC, PSCC, and anterior semicircular canal (ASCC) on the affected side. In another case, vHIT results showed reduced VOR gain combined with CuS in the HSCC and PSCC on the affected side. Six of the eight patients with bilateral hypofunction of CaT had negative SVINT results, two of whom had normal vHIT results, two of whom had vHIT results that showed reduced VOR gain combined with CuS in the bilateral PSCC, and two of whom had vHIT results that showed reduced VOR gain combined with CuS in the PSCC on the affected side. Six of 16 patients with normal CaT had positive SVINT results. Two of them had normal vHIT results, two had reduced VOR gain combined with CuS in the HSCC on the affected side, and two had reduced VOR gain combined with CuS in the PSCC on the affected side. The two detection methods, CaT and SVINT, showed fair agreement in identifying vestibular asymmetry (*kappa* = 0.324, *p* = 0.005 < 0.05). There was no statistically significant difference in the positive rates between the two methods (*McNemar*, *p* = 0.21 > 0.05).

### 3.3 SVINT vs. vHIT

The distribution of SCC impairment in vHIT and SVINT results is shown in Table 2. Thirty-three patients (75%) exhibited reduced VOR gain and CuS on the affected side, and they also had a positive SVINT result. Six patients (13.6%) had reduced VOR gain combined with CuS in unilateral HSCC, 13 patients (29.5%) had reduced VOR gain combined with CuS in unilateral HSCC and PSCC, and 2 patients (4.5%) had reduced VOR gain combined with CuS in unilateral HSCC and ASCC. Nine patients (20.5%) had reduced VOR gain combined with CuS simultaneously in unilateral HSCC, PSCC, and ASCC on the affected side. Eleven patients (25%) had reduced VOR gain combined with CuS only in PSCC on the affected side, of which four cases had positive SVINT results. However, the SPV of nystagmus on the affected side was significantly greater than on the healthy side in three of these four cases. Bilateral PSCC with reduced VOR gain with CuS were also observed in 3 cases, all of which showed negative SVINT results. Compared with impairment of only the horizontal semicircular canal or simultaneous impairment of the horizontal and vertical semicircular canals, the positive rate of SVINT was lower when only the posterior semicircular canal was impaired, and the difference was statistically significant (*F* = 21.195, *p* < 0.05).

**Table 2:** Distribution of SCC impairment in vHIT and positive SVINT.

SCC impairment in vHIT	Patients (*n*)	Positive SVINT	
		*n*	%
Unilateral HSCC (*n* = 6)	6	6	100
Unilateral HSCC and PSCC (*n* = 13)	13	12	92.3
Unilateral HSCC and ASCC (*n* = 2)	2	2	100
Unilateral HSCC, ASCC, and PSCC (*n* = 9)	9	9	100
Unilateral PSCC and bilateral PSCC (*n* = 14)	14	4	28.6
Total	44	33	75

n = number of patients; Fisher’s exact test (*F*) was used for statistical analysis.

In vHIT results, the abnormal rates of PSCC (50.7%) were the highest, followed by the HSCC (42.3%) and ASCC (15.5%). The abnormal rate of both PSCC and HSCC was significantly higher than that of ASCC, and the difference was statistically significant (*χ^2^* = 19.877, *p* < 0.0125) (*χ^2^* = 12.379, *p* < 0.0125).

### 3.4 Correlation of SVINT with CaT and vHIT

Calculations regarding SVINT characteristics showed better agreement between SVINT and vHIT-HSCC results (*kappa* = 0.668, *p* < 0.05) than that between SVINT and other tests (Table 3). When the vHIT-HSCC results were used as a standard, it had a sensitivity of 96.7%, a specificity of 73.2%, a positive predictive value of 72.5%, and a negative predictive value of 96.8%. The SVINT and vHIT-HSCC show a certain degree of agreement in assessing vestibular asymmetry, but there is a statistically significant difference in their positive rates (*McNemar*, *p* < 0.05), with SVINT having a higher false positive rate. In practical applications, it is essential to be vigilant about the risk of overdiagnosis while clearly defining its appropriate use cases—specifically for initial screening rather than definitive diagnosis.

**Table 3:** Correlation of SVINT with CaT and vHIT results.

SVINT	Positive CaT	Positive vHIT	Positive vHIT-HSCC	Positive vHIT-VSCC
Positive SVINT	32	33	29	28
Negative SVINT	15	11	1	10
Total	47	44	30	38
Kappa	Kappa = 0.324 *P* < 0.05	Kappa = 0.477 *P* < 0.05	Kappa = 0.668 *P* < 0.05	Kappa = 0.345 *p* < 0.05
McNemar	*P* > 0.05	*P* > 0.05	*P* < 0.05*	*P* > 0.05

Positive CaT indicates unilateral hypofunction; *p* < 0.05* indicates a higher positive rate for SVINT (56.3%) compared to vHIT-HSCC (42.3%).

### 3.5 SVINT and audiogram types and degree of hearing loss

The patients in the SSNHL-V group contained one case of low-frequency hearing impairment, 8 cases of high-frequency hearing impairment, 26 cases of flat hearing impairment, and 36 cases of full-frequency hearing impairment. The differences between the positive rates of SVINT, CaT, and vHIT results and the hearing curve classifications were not statistically significant (Table 4). The patients in the SSNHL-V included 3 cases of mild hearing impairment, 8 cases of moderate hearing impairment, 21 cases of severe hearing impairment, and 39 cases of profound hearing impairment. The difference between the degree of hearing impairment and the positive rate of SVINT, CaT, and vHIT results showed no significant differences (Table 4).

**Table 4:** Abnormal rate of SVINT, CaT, and vHIT in different audiogram types and hearing loss.

Audiometric Results	Positive CaT		Positive vHIT		Positive SVINT	
	*n*	%	*n*	%	*n*	%
Audiogram type						
Low-frequency hearing loss (*n* = 1)	1	100	0	0.0	0	0.0
High-frequency hearing loss (*n* = 8)	6	75	5	62.5	3	37.5
Flat hearing loss (*n* = 26)	14	53.8	14	53.8	12	46.2
Full-frequency hearing loss (*n* = 36)	26	72.2	25	69.4	25	69.4
Classification of hearing loss						
Mild–moderate hearing loss (*n* = 11)	6	54.5	5	45.4	6	54.5
> severe hearing loss (*n* = 60)	41	68.3	39	66	41	68.3

There were no significant differences in the abnormal SVINT, CaT, and vHIT rates among the four audiogram types. SVINT (*F* = 5.968, *p* = 0.75), CaT (*χ*^2^ = 3.144, *p* = 0.411), vHIT (*χ*^2^ = 1.574, *p* = 0.495). There were no significant differences in the abnormal SVIN, CaT, and vHIT rates among the degrees of hearing loss. SVINT (*χ*^2^ = 0.017, *p* = 0.896), CaT (*χ*^2^ = 0.790, *p* = 0.374), vHIT (*χ*^2^ = 1.507, *p* = 0.22). Positive CaT indicates unilateral hypofunction.

## 4 Discussion

### 4.1 Correlation between SVINT and vHIT

The agreement between SVINT and HSCC results of vHIT was most significant in patients with SSNHL-V in our study. Our findings further suggest that SVINT responses in patients with SSNHL-V are dominated by the high-frequency functional asymmetry of the HSCC and are less sensitive to the low-frequency functional asymmetry of the HSCC as well as to abnormalities of the vertical SCC.

The SVINT detects vestibular function asymmetry of either side more sensitively in response to peripheral than central abnormalities (17). Its clinical applications in vestibular schwannomas (VS), Meniere’s disease (MD), vestibular neuritis (VN), and Ramsay-Hunt syndrome have been described and validated. According to Dumas et al. (22), 75% of 25 VS patients exhibited severe unilateral hypofunction on CaT results, and 64% could elicit positive SVINT results. Nuti and Mandala (23) reported that 75% of 28 VN patients induced a positive SVINT result, and 93% had a unilateral hypofunction of CaT. Fifty-two MD patients during the irritative phase were examined by Hong et al. (24), and 71% of patients had a positive SVINT result. They concluded that SVINT results were related to the severity of CaT hypofunction (24). Kim et al. (25) compared the positive rates of SVINT results in patients with Ramsay-Hunt syndrome, SSNHL-V, and VN. Among patients with CP > 25% on the CaT, 91% of VN patients, 89% of patients with SSNHL-V, and 94% of patients with Ramsay-Hunt syndrome were able to induce a positive SVINT result (25).

As mentioned above, most research assessing SVINT and vestibular function asymmetry has focused on the level of asymmetry in CaT results. Although there was a significant difference in stimulation frequency between CaT and SVINT, there was some correlation between SVINT and CaT, which is consistent with our findings. However, in our comparison of the three tests, SVINT, CaT, and vHIT, we found that the agreement between SVINT and vHIT was superior to that between SVINT and CaT. This may be related to the “dual frequency” theory of vestibular hair cells. Vestibular hair cells of type I are susceptible to high-frequency stimulation, whereas those of type II are sensitive to low-frequency stimulation (26, 27). CaT primarily involves type II hair cell receptors, which are activated by regular vestibular afferent neurons that respond to low-frequency and low-acceleration stimuli to the vestibular system. Irrigation at different temperatures creates a temperature gradient across the HSCC of the irrigated ear, which alters the density of the endolymph, causing it to flow under gravity and deflect the ampulla, resulting in a non-physiological stimulus. Moreover, CaT is still the only vestibular test capable of isolating the stimulated side (28). The vHIT initiation response may arise from type I vestibular hair cell receptors and their irregular vestibular afferent (29). The SVINT stimulates both labyrinths simultaneously, causing displacement of the endolymph. Type I hair cells located at the crest of the crista or the striola of the maculae activate the irregular vestibular afferent neurons. Conversely, at somewhat clinically safe levels, regular vestibular afferents activated by type II hair cell receptors had little to no response to vibration stimuli (30).

The vHIT evaluates the high-frequency function of all six SCCs. When the HSCC results of vHIT were used as a criterion, the sensitivity and specificity of SVINT in identifying the HSCC asymmetry were better than those in identifying vertical SCC asymmetry. This result further suggests that the response of SVINT is primarily driven by the HSCC, which is in accordance with what was reported by Fabre et al. (31). They evaluated the correlation between SVINT’s different components and other vestibular tests with corresponding vestibular receptor structures in 52 patients with peripheral vestibular disorders. It was concluded that the horizontal component of SVINT was predominantly related to the HSCC and utricle (31). Moreover, their report and this study observed no correlation between the PSCC results of vHIT and SVINT. Dumas et al. (32) found that the contribution of HSCC dominated the SVINT results, followed by the utricle, after comparing the SVINT results of 40 patients with superior semicircular canal dehiscence syndrome (SSCDS) and 18 patients with severe unilateral vestibular loss.

### 4.2 Audio-vestibular correlations in SSNHL patients

The pathogenesis of SSNHL is complex and multifactorial, often involving microcirculatory disturbances in the inner ear, which can subsequently impair cochleovestibular function. It is important to note that both central vestibular structures and peripheral vestibular structures share a common vascular supply—branches of the vertebrobasilar artery. Therefore, ischemic infarcts or hemorrhages in the vertebrobasilar territory may present with varied clinical manifestations depending on the affected anatomical structures or exhibit overlapping symptoms due to shared vascular territories (33). Among these, hearing loss accompanied by vertigo may also serve as a prodromal symptom of AICA stroke. The HINTS Plus protocol is typically recommended as a bedside test battery for the early identification of stroke, with high sensitivity and superior to MRI in the initial diagnosis (8).

The vHIT results in this paper show a higher abnormality rate in PSCC than in other SCCs in patients with SSNHL-V. This finding aligns with earlier discoveries. In analyzing vestibular function test findings from 71 patients with SSNHL, Lee et al. (34) observed that PSCC impairment was most common in patients with spontaneous nystagmus. Pogson et al. (35) reported that 74% of 27 patients with SSNHL-V were seen to have a reduced VOR gain (0.45 ± 0.20) in the PSCC, with a higher rate of PSCC abnormalities compared to the other SCCs. On the one hand, relevant studies have suggested that this phenomenon may be linked to the labyrinth’s vascular supply. The anterior vestibular artery, a branch of the labyrinthine artery, supplies the HSCC, ASCC, the superior part of the saccular macula, and the utricular macula (36). However, the posterior vestibular artery, a branch of the common cochlear artery, supplies the PSCC and part of the saccular macula (37). The posterior vestibular arteries have no collaterals, which makes them more vulnerable to vascular damage. In particular, damage to the common cochlear artery may lead to SSNHL and isolated PSCC hypofunction. A group of cases with this specific pattern of lesions in the cochlea and PSCC was described by Rambold et al. (38). Animal studies have shown that 30 min or more of ischemia results in irreversible damage (39). The vascular supply and the proximity of the nerve fibers provide plausibility for this result. A filling defect in the PSCC was identified in an inner ear MRI 3D-FIESTA of a patient with left-sided SSNHL-V, consistent with ischemia-induced fibrosis of the PSCC (40), which again provides clues to the theory of vascular supply. On the other hand, the PSCC and saccule are innervated by the inferior vestibular nerve, which is positioned close to the cochlear nerve. Thus, functional impairment in PSCC may be more relevant to the severity of the cochlear injury.

Patients with SSNHL who experience vertigo have been found to have a link between hearing loss and vestibular impairment. CaT findings for 135 SSNHL patients were reported by Shih et al. (41). They discovered that abnormal CaT results were highly correlated with the severity of hearing impairment, accompanying vertigo, and poor recovery (41). In a logistic regression analysis of 156 patients with SSNHL-V, Wang et al. found that the odds of having vestibular dysfunction in profound hearing loss were 3.89 times higher than in patients with low-frequency hearing loss (42). However, our investigation revealed that SVINT results in patients with SSNHL-V did not correlate with the degree of hearing loss and the audiogram type. The small sample size in this article could be a contributing factor. The existing study on audio-vestibular correlations in peripheral disorders remains controversial, with either no correlation (35, 43) or a weak correlation (44, 45) being found, indicating that further research in this area is still necessary.

## 5 Limitations

To better evaluate the ability of SVINT to detect the asymmetry of vestibular function in patients with SSNHL-V, it is necessary to test a larger sample of cases to validate our observations. This article does not address the assessment of otolith function. Both the SCCs and the otolith organs are involved in the SVINT response; the proportion of each component’s contribution is not precise, and the results of VEMP should be included in further study designs.

## 6 Conclusion

Unilateral SSNHL-V may be accompanied by vestibular dysfunction of varying degrees, and vestibular function may be shown as asymmetrical results of CaT and/or vHIT, as well as asymmetrical otolith function. SVINT may provide a rapid identification and screening of HSCC high-frequency functional asymmetry in patients with SSNHL-V. However, SVINT has limited clinical application regarding disease localization and bilateral vestibular symmetry impairment. Other test results, such as CaT, SOT, vHIT, and VEMP, need to be referenced when interpreting the SVINT results.

## Data Availability

The original contributions presented in the study are included in the article/Supplementary material, further inquiries can be directed to the corresponding authors.
