# Correction: Correlation of skull vibration-induced nystagmus test and video head impulse test in patients with sudden sensorineural hearing loss with vertigo

**DOI:** 10.3389/fneur.2025.1714102

**Published:** 2025-10-14

**Authors:** Qiang Guo, Ying Lin, Pengfei Hang, Dingjun Zha

**Affiliations:** Department of Otolaryngology, Head and Neck Surgery, Xijing Hospital, Air Force Medical University, Xi'an, China

**Keywords:** skull vibration-induced nystagmus test, sudden sensorineural hearing loss, hearing audiogram, video head impulse test, caloric test

The funder [Innovative Research Team on the Fundamentals and Clinical Applications of Sensorineural Hearing Loss], [2023-CX-TD-70] to [Qiang Guo] was erroneously omitted.

The funder [Shaanxi Provincial Health Science Research and Innovation Platform for Otorhinolaryngology], [2024PT-07] to [Qiang Guo] was erroneously omitted.

The funder [Research on Key Technologies for the Prevention and Control of Noise-Induced Hearing Loss], [2024SF-ZDCYL-01-16] to [Qiang Guo] was erroneously omitted.

The correct funding statement reads:

The author(s) declare that financial support was received for the research and/or publication of this article. This work was supported by Innovative Research Team on the Fundamentals and Clinical Applications of Sensorineural Hearing Loss (grant number: 2023-CX-TD-70), Shaanxi Provincial Health Science Research and Innovation Platform for Otorhinolaryngology (grant number: 2024PT-07), and Research on Key Technologies for the Prevention and Control of Noise-Induced Hearing Loss (2024SF-ZDCYL-01-16) to QG.

The original version of this article has been updated.

